# Microsatellite instability in gastric cancer: molecular bases, clinical perspectives, and new treatment approaches

**DOI:** 10.1007/s00018-018-2906-9

**Published:** 2018-09-01

**Authors:** Margherita Ratti, Andrea Lampis, Jens C. Hahne, Rodolfo Passalacqua, Nicola Valeri

**Affiliations:** 1 Division of Molecular Pathology, The Institute of Cancer Research, London, UK; 2 Division of Oncology, Medical Department, ASST di Cremona, Ospedale di Cremona, Cremona, Italy; 3 Department of Medicine, The Royal Marsden NHS Foundation Trust, London, UK

**Keywords:** Microsatellite instability, Gastric cancer, Molecular stratification, Predictive and prognostic value, Adjuvant chemotherapy, Immune-checkpoint inhibitors

## Abstract

Gastric cancer is one of the most aggressive malignancies, with limited treatment options in both locally advanced and metastatic setting, resulting in poor prognosis. Based on genomic characterization, stomach tumour has recently been described as a heterogeneous disease composed by different subtypes, each of them with peculiar molecular aspects and specific clinical behaviour. With an incidence of 22% among all western gastric tumour cases, stomach cancer with microsatellite instability was identified as one of these subgroups. Retrospective studies and limited prospective trials reported differences between gastric cancers with microsatellite stability and those with instability, mainly concerning clinical and pathological features, but also in regard to immunological microenvironment, correlation with prognostic value, and responses to treatment. In particular, gastric cancer with microsatellite instability constitutes a small but relevant subgroup associated with older age, female sex, distal stomach location, and lower number of lymph-node metastases. Emerging data attribute to microsatellite instability status a favourable prognostic meaning, whereas the poor outcomes reported after perioperative chemotherapy administration suggest a detrimental role of cytotoxic drugs in this gastric cancer subgroup. The strong immunogenicity and the widespread expression of immune-checkpoint ligands make microsatellite instability subtype more vulnerable to immunotherapeutic approach, e.g., with anti-PD-L1 and anti-CTLA4 antibodies. Since gastric cancer with microsatellite instability shows specific features and clinical behaviour not overlapping with microsatellite stable disease, microsatellite instability test might be suitable for inclusion in a diagnostic setting for all tumour stages to guarantee the most targeted and effective treatment to every patient.

## Introduction

**Fig. 1:**
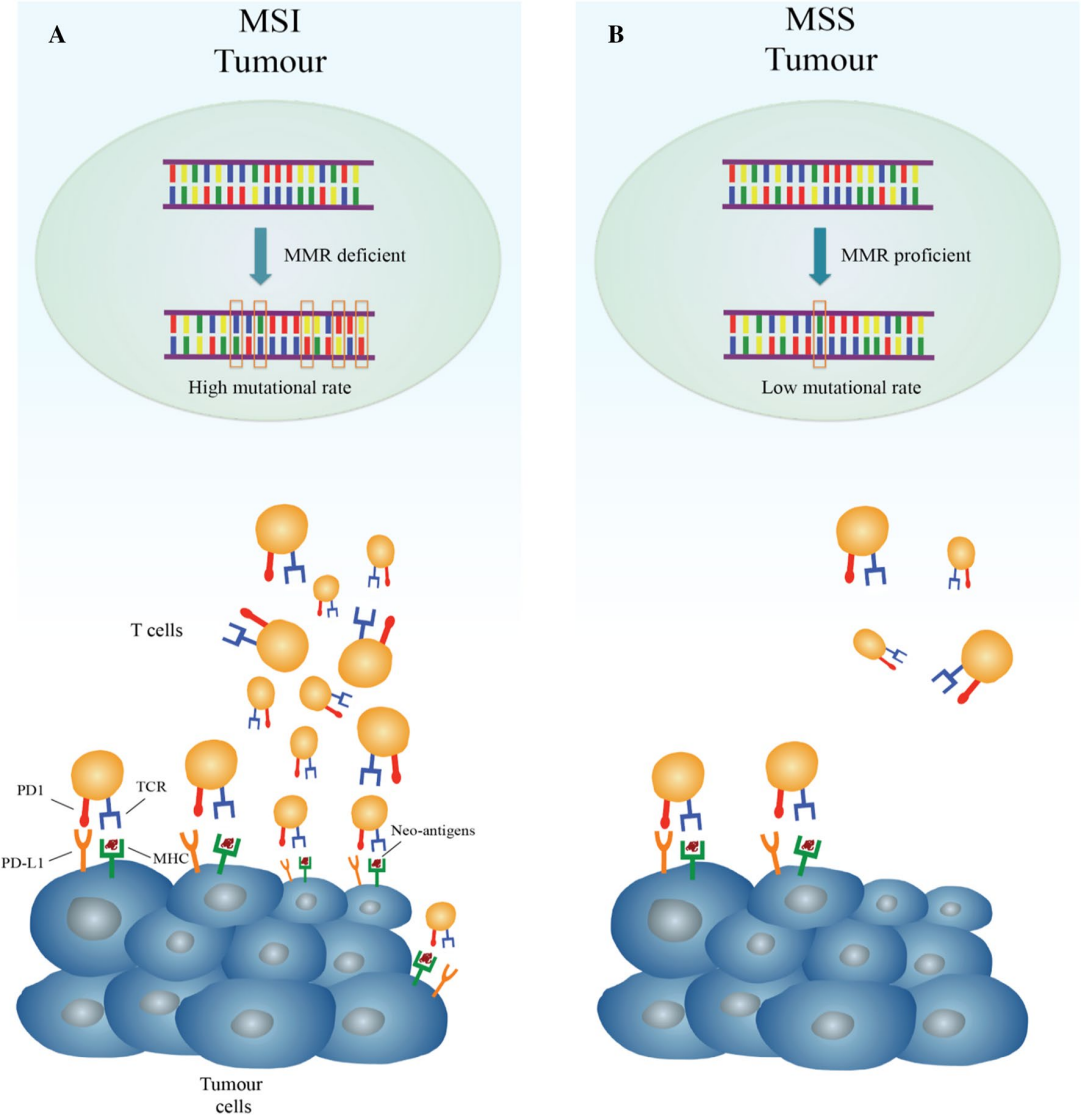
Different immune microenvironment in microsatellite instability-high (MSI) hypermutated tumours and in microsatellite stable (MSS) tumours with low-mutational rate. **a** In the presence of deficient mismatch repair (MMR), DNA replications errors go undetected and unrepaired, leading to a tumour with high mutational burden. Hyper-mutated cancer cells produce several neo-antigens, which stimulate T-cell activation and tumour infiltration by immune cells. To counteract this vigorous immune response, tumour cell exposes checkpoint molecules, e.g., PD-L1, to inhibit anti-tumour activity. **b** In the presence of functional MMR system, replication errors occur rarely with lower mutational rate and, as a consequence, limited production of neo-antigens. For this reason, in MSS tumour, the amount of T-cell infiltration and checkpoint molecules exhibition is low. The peculiar immune microenvironment of MSI tumours is thought to explain why they are ideal target for therapy with immune-checkpoint inhibitors. *MHC* major histocompatibility complex, *TCR* T-cell receptor

Gastric cancer (GC) is one of the most common tumours and the third leading cause of cancer-related death worldwide [1]. The addition of targeted drugs to the established chemotherapeutic scenario of treatment has determined a modest improvement in overall survival, but, unfortunately, the prognosis remains poor [2–4]. Emerging data suggest that patients’ outcomes do not only depend on staging but also on specific molecular and histopathologic tumour features [5, 6]. Indeed, two detailed genomic characterizations of gastric cancer have recently been developed by The Cancer Genome Atlas (TCGA) and the Asian Cancer Research Group (ACRG) [5, 6], proving that GC is a complex and heterogeneous disease. According to TCGA genomic characterizations, GC can be divided into four subgroups (Table 1): (1) tumours positive for Epstein–Barr Virus (EBV) infection; (2) microsatellite instability-high tumours (MSI-H); (3) genomically stable tumours (GS); (4) tumours with chromosomal instability (CIN) [5, 6]. Interestingly, the MSI-H subgroup was identified as a separate entity of GC in both of these classifications [5, 6], with a reported incidence in the western population of 22% [5]. The frequency of MSI across gastrointestinal cancers and tumours of other districts with high prevalence of MSI (> 10%) are summarized in Table 2. Microsatellites are short and repetitive DNA sequences randomly widespread throughout the genome [5, 7, 8]. The mismatch repair system deficiency (MMRD) is generally caused by germline mutations or sporadic epigenetic silencing that lead to insertion or deletions of nucleotides in the microsatellite regions during DNA replication; these phenomena are known as microsatellite instability (MSI) [9–11]. Although the role of MSI-H in colorectal cancer as a predictive and prognostic factor is well established [12–16], the correlation between MSI, and clinical and pathological features in GC remains ambiguous, with a few available data from prospective trials [17–20]. Interestingly, recent studies have hypothesized that alterations in the mismatch repair (MMR) system may predict clinical benefit for treatment with immune-checkpoint inhibitors, due to a positive correlation between MSI-H and PD-L1 expression, as shown in Fig. 1 [21–24]. In this review, the current evidences about microsatellite instability-high (MSI-H) gastric cancer (GC) are summarized, with a special focus on pathological characteristics, predictive and prognostic values, and future perspectives for clinical approaches of MSI-H GC subgroup.

**Table 1:** Four gastric cancer subtypes, as described by TCGA, with reported frequency and main histological and molecular features

TCGA gastric cancer subgroups	Frequency (%)	Main characteristics
Epstein–Barr virus (EBV)	9	Gastric fundus location CDKN2A silencing Hypermethylation of CpG islands Over-expression of immune-checkpoint ligands
Microsatellite instability (MSI)	22	Body and pyloric gastric location Correlation with Lauren intestinal subtype Hypermutation status MLH1 silencing and hypermethylation of CpG islands
Genomically stable (GS)	20	Homogenous distribution to all portions of the stomach Correlation with Lauren diffuse histology CDH1 and RHO mutations, CLDN18–ARHGAP fusion
Chromosomal instability (CIN)	49	Homogenous distribution to all portions of the stomach Correlation with Lauren intestinal histology Activation of RAS pathway Mutation of TP53

**Table 2:** Percentage of MSI frequency in gastrointestinal and non-gastrointestinal cancers with high prevalence (≥ 10%) of MSI status

Tumour site	MSI frequency (%)	Study	
Colorectal cancer	15	Poynter et al. [68]	MSI frequency across gastrointestinal tumours
Hepatocellular carcinoma	10–43	Karachristos et al. [69] Chiappini et al. [70]	
Gastric cancer	10–22	Kim et al. [44] The Cancer Genome Atlas Network [5]	
Intrahepatic cholangiocarcinoma	10	Silva et al. [71]	
Duodenal and ampullary carcinoma	10	Achille et al. [72] Ruemmele et al. [73] Agaram et al. [74] Achille et al. [75]	
Esophageal adenocarcinoma	7	Farris et al. [76]	
Gallbladder cancer	0–42	Silva et al. [71] Yoshida et al. [77]	
Pancreatic adenocarcinoma (ductal)	0–13	Yamamoto et al. [78] Laghi et al. [79]	
Endometrial cancer	22–33	Zighelboim et al. [80] Aguirre et al. [81]	Non-gastrointestinal tumours with higher MSI frequency (≥ 10%)
Sebaceous skin cancer	20–25	Cesinaro et al. [82] Kruse et al. [83]	
Ovarian cancer	10	Jensen et al. [84] Segev et al. [85]	
Thyroid cancer	0–63	Stoler et al. [86] Bauer et al. [87]

## Microsatellite instability and the mismatch repair genes system

Microsatellites are DNA sequences with a length ranging from one to six repetitions of nucleotides (usually between 10 and 60 times) [25]. These DNA motifs are scattered throughout coding and non-coding regions of the genome, highly polymorphic among population but stable in each individual [25]. The MMR system consists of several proteins, which include the products of *hMLH1*, *hMSH2*, *hMSH6*, and *hPMS2* genes, which are responsible for surveillance of correct DNA replication. The MMR system targets and corrects replication errors (e.g., base mismatch, insertions, and deletions) when detected [26–28]. The heterodimeric protein complexes hMSH2/hMSH6 and hMSH2/hMSH3 are responsible for the initial detection of replication errors. The subsequent recruitment of the complex formed by hMLH1 and hPMS2 removes the mismatched nucleotide or fragment and allows DNA re-synthesis [28]. Inactivation of MMR proteins can be caused by mutations in the coding region, promoter methylation, or chromosomic rearrangements that lead to loss of heterozygosity [28–30]. Microsatellite unstable GC can be observed in sporadic GC and in the setting of Lynch syndrome [11, 29, 30]. Lynch syndrome is caused by autosomal dominant mutations in the MMR genes—mainly *hMLH1* and *hMSH2* and less frequently *hPMS2* and *hMSH*6 [30]. Moreover, a constitutional 3′-end deletion of *EPCAM*, which is immediately upstream of the *MSH2* gene, may cause Lynch syndrome through epigenetic silencing of *MSH2* [31]. Patients affected by Lynch syndrome present an increased predisposition to develop colorectal cancer and endometrial cancer, but also to ovarian and gastric cancer occurring at a younger age (11.3-fold in the 30s and 5.5-fold in the 40s) [28–30]. Increased risk for developing pancreatic, bladder and breast cancer, and most possibly also prostate cancer has been related with Lynch syndrome carriers [31]. Patients with *MSH6* mutations appear to be particularly at risk of gastrointestinal and endometrial cancers, whereas carriers of an *MSH2* gene mutation have the highest cancer risks across the spectrum, especially for the development of urinary tract cancer [31]. In the sporadic setting, more than 50% of MSI GCs contain an epigenetic hypermethylation of *hMLH1* promoter, whereas mutations in *hMLH1* and *hMSH2* have been reported in 12–15% of this GC subgroup [32]. Gene expression inactivation by alternative unknown genetic or epigenetic alterations have been hypothesized to be responsible for all of the remaining cases of microsatellite unstable GC [32]. The functional loss of MMR proteins results in a highly mutated phenotype with a large number of frameshift and missense mutations in key oncogenes and tumour suppressor genes. Mutations in genes responsible for cell cycle regulation and apoptosis (e.g., *TGFβ RII*, *IGFIIR*, *TCF4*, *RIZ*, *BAX*, *CASPASE5*, *FAS*, *BCL10*, and *APAF1*) or for genomic integrity maintenance (e.g., *hMSH6*, *hMSH3*, *MED1*, *RAD50*, *BLM*, *ATR*, and *MRE11*) have been also associated with MSI-H GC [11]. Moreover, increased expression of mitotic pathways components, such as AURKA A/B, E2F, FOXM1, PLK1, and targets of MYC activation, has been described and confirmed on a transcriptomic level in MSI-H tumours [5]. Indeed, inactivation of MMR genes is not, by itself, a transforming event and additional genetic changes are needed to determine tumour progression. It is well established that MSI cancers are associated with 100- to 1000-fold increased mutation rates compared to microsatellite stable (MSS) tumours [11, 29, 30, 33]. The repetitive sequences of microsatellites are particularly prone to replication errors, and therefore, they can be used as a marker for an intact or defective MMR system [11].

## Diagnosis of MSI

The increasing knowledge about the prognostic and predictive role of MSI-H vs MSS in several cancer subtypes has led to a larger number of patients routinely tested for this molecular feature [32]. Gastrointestinal and non-gastrointestinal cancers with high prevalence of MSI-H (≥ 10%) are summarized in Table 2. For an accurate determination of MSI status and the subsequent therapeutic decision, sensitive, fast, and precise techniques are necessary [30–32]. Currently, several different methods are validated and in use to detect an MMR deficient cancer:

1. polymerase chain reaction (PCR) amplification of microsatellite sequences;
2. immunohistochemistry (IHC) staining for expression of MMR proteins;
3. next-generation sequencing (NGS) for detection of MSI.

### MSI evaluation by polymerase chain reaction (PCR)

**Fig. 2:**
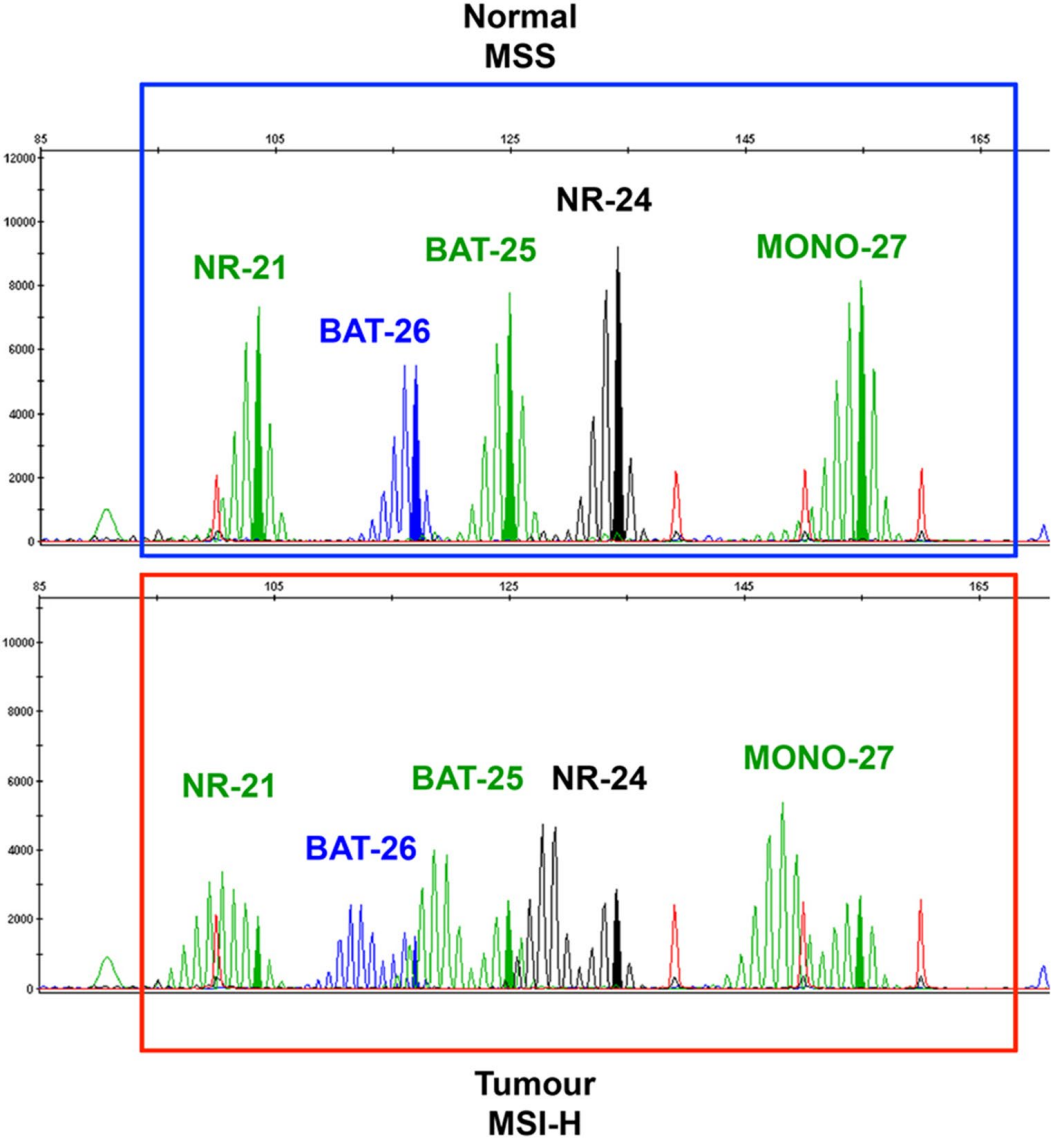
Representative capillary electrophoresis (pherogram) of the Promega MSI Analysis System generated using GeneMapper 3.7 Analysis Software. The upper part of the figure shows microsatellite stability (MSS) in normal tissue, without shifted alleles. The lower part is representative of tumour microsatellite instability-high (MSI) in all *loci,* with evident alleles shifting. Green: peaks of mononucleotides NR-21, BAT-25, and MONO-27. Blue: peak of BAT-26. Black: peak of NR-24

PCR amplification with specific primers for microsatellite repeats results in a distinctive amplification profile [30, 34]. By comparing the allelic position of the microsatellite locus in tumour and normal tissue, MSI can be assessed as “shift” in the pherogram of one or more microsatellites as illustrated in Fig. 2. To reach high specificity and sensitivity and also to ensure reproducibility and standardization between different laboratories, The National Cancer Institute recommends the so-called Bethesda Panel as reference for diagnostic testing [7]. This panel is composed of five microsatellite markers specific for two mononucleotide loci (BAT-25 and BAT-26) and three dinucleotide loci (D2S123, D5S346, and D17S250) [7, 29]. These regions are amplified in parallel using fluorescent PCR and their sizes are evaluated by subsequent capillary electrophoresis [34]. Using this method, three different *stati* can be established based on different allelic size patterns in the cancer tissue compared to the normal one. The MSI-high (MSI-H) status is given by a shift in size in at least two of the five microsatellite loci; MSI-low (MSI-L) is given by a shift in size in one locus out of five and microsatellite stable (MSS) with any shift in cancer tissue compared to the normal one [7, 30, 34]. The dinucleotide markers were demonstrated to be less sensitive and specific than mononucleotide for the detection of tumours with mismatch repair deficiencies [35]. Furthermore, mononucleotide markers are more commonly quasi-monomorphic, potentially obviating the need to test the corresponding normal DNA. [7]. To overcome the limitations of Bethesda system due to the presence of dinucleotide markers, a commercial available panel has recently been developed by Promega Corporation. This is commonly employed in the diagnostic practice and replaced dinucleotides of the Bethesda Panel with mononucleotide markers (NR-21, NR-24, and MONO-27, see Fig. 2) helping to resolve cases of MSI-low into either MSI-H or MSS by comparison of tumour and the surrounding normal tissue as illustrated in Fig. 2 [7, 35].

### MSI evaluation by immunohistochemistry (IHC)

Immunohistochemistry staining allows detection of expression or total absence of MMR proteins and relative scoring is possible. This method shows comparable performance characteristics and high concordance rate (> 90%) with MSI detection with PCR [8]. The loss of expression of a single protein or of a heterodimeric couple of the MMR complex suggests the presence of MMRD; thus, it is an indirect evidence of MSI. On protein level, hMLH1 and hMSH2 are stable without their respective dimeric partners’ hPMS2 and hMSH6, whereas these latter components are rarely stable without their counterparts [17, 28, 30]. Therefore, tumours with mutated *hMLH1* or *hMSH2* genes usually show loss of the respective functional dimer; conversely, mutations of *hPMS2* or *hMSH6* genes generate loss of only the affected protein [28, 30]. Hence, IHC allows the determination which of the MMR genes is defective and supports the decision about further genetic analysis [30, 34]. It must be taken into account that IHC provides misleading information for those rare cases of missense mutations in *hMLH1* or *hMSH6* genes, resulting in translated proteins with normally antibody affinity but missing enzymatic activity. In these cases, only PCR-based MSI testing can help to determine whether there are true functional MMR proteins through these mutations [7, 11].

### Comparison between MSI testing via PCR and IHC

Many studies have attempted to evaluate and compare the best and cost-effective method in defining the MMR status between IHC and PCR [7, 8, 36]. Moreover, it has been evidenced in many reports the high correlation between IHC results and PCR-based tests in determining the phenotypic trait of the tumour [7, 36]. In a recent study, a discrepancy between MMRD and MSI assessment was found [20]. Nevertheless, the overall concordance between immunohistochemical analysis of MMR protein expression and MSI was high [20]. The authors explained the imperfect correlation with interobserver variability in immunohistochemical analysis assessment, heterogeneity of biomarker expression in gastric cancer, and the presence of normally translated but non-functional MMR proteins in the setting of a missense MLH1 mutation, or rare genomic defects that result in MSI-H status with intact MMRD function, e.g., polymerase DNA ε1 mutation [20].

One of the advantages of IHC technique consists in its wide integration in routine testing in molecular and diagnostic pathology laboratories and in its ability in identifying which gene should be investigated for further molecular analyses in case of suspected hereditary cancer syndromes [30, 36]. Moreover, when IHC is used only the tumour tissue is required, whereas both normal and tumour samples are required for MSI testing with PCR [7]. Molecular testing with PCR detects MSI directly as a consequence of MMRD. In these 5–11% of MSI malignancies that do not exhibit MMR protein loss, usually due to retained antigenicity in an otherwise non-functional protein, IHC may underestimate MSI-H cases. In this situation, PCR-based test helps defining the correct diagnosis [20, 34, 36].

### MSI evaluation by next-generation sequencing (NGS)

Some laboratories started to use NGS to diagnose the microsatellite status [37–39]. In most cases, also NGS-based MSI determination needs paired tumour and normal tissue [38, 39]. Recently, a MSI assay that uses data from a commercial available NGS panel for determination of the MSI status has been established [37]. One advantage of this NGS-based assay for MSI evaluation is that it does not require matched samples from normal tissue. Furthermore, NGS-based methods cover a broader range of microsatellite loci; thus, it is not limited to the five microsatellite sites used in the PCR-based method [37]. The disadvantages are the high investment costs per sample for NGS and the longer time needed to perform NGS run and bioinformatic analysis in comparison to PCR and IHC-based MSI analysis methods.

## Clinico-pathological features of MSI gastric cancer

The highest incidence of GC is reported amongst Asiatic population [1], and thus, it is not unexpected that most of the information about histologic and clinical characteristics of microsatellite unstable GC is based on retrospective studies involving Asian GC patients [40–43]. Furthermore, MSI-H GC prevalence in Asians is commonly < 10% of all GC cases [44], lower than most of the rates reported in the Western studies concerning this topic [5, 45, 46]. Many data have been collected and analyzed to clarify whether MSI-H could be considered as a separate GC subgroup with specific histopathologic features, clinical behaviour, and different response to chemotherapy and immunotherapy. TCGA first provided a comprehensive molecular characterization of GC based on 295 cases from a western population, and categorized them into four different subtypes (Table 1) [5]. The GC MSI-H subgroup is characterized by elevated mutation rate in genes encoding proteins involved in oncogenic signalling pathways, mutations at “hotspot” regions such as *PIK3CA*, *ERBB3*, *ERBB2,* and *EGFR* genes already described in other malignancies, e.g., colon and breast cancers, as well by methylation at the *hMLH1* gene promoter [5, 47, 48]. Interestingly, in microsatellite unstable GC, BRAF V600E mutation, frequently reported in sporadic colon cancer caused by MSI, has never been described [47]. In general, MSI-H GC is associated to older age (> 65-year-old patients), female sex, onset in the distal stomach, intestinal type (according to Lauren classification) and more common in patients with multiple synchronous gastric cancers than in those with a solitary tumour [49]. The examination of multiple early GC treated with endoscopic mucosal resection revealed that MSI-H status increased the frequency of both synchronous and metachronous GC [50]. Among all these features, the association between onset at older age and MSI-H phenotype is observed in most of the studies focusing on this topic [40, 42, 44–46]. Methylation of *hMLH1* gene and its progressive loss of expression have been related to aging [51]. Methylation of this gene is the main cause for microsatellite unstable status in sporadic GC; this might explain the connection between onset of MSI-H GC and aging [29, 30]. Interestingly, in many studies, this subgroup showed a specific phenotype sharing similarities with medullary-type cancer or presenting lympho-plasmacytoid appearance [46]. More precisely, it was observed that MSI-H GC was enriched with highly pleomorphic tumour cells arranged in several growth patterns surrounded by an inflammatory stroma, with pushing tumour borders and widespread expression of immune-checkpoint ligands [45, 46]. Many studies showed a positive correlation between the intestinal subtype and the microsatellite unstable phenotype, whereas poorly cohesive and diffuse histology are rarely associated with this GC subgroup. These findings are not always statistically robust, probably due to the small sample sizes involved in these studies [41, 44, 45]. Moreover, in some reports, an association between MSI-H and tumour phenotype was not found [45, 46]. In clinical setting, patients with MSI-H GC show a significant longer overall survival (OS) compared with those who have GC with MSS GC features. It has been argued that MSI-H GC has a better prognosis due to its correlation with earlier TNM stage at diagnosis (stages I–II), limited lymph-node metastasis, and Lauren intestinal histotype [45, 46]. Moreover, immunological assessment of the microenvironment in MMRD tumours exhibits enhanced attraction of tumour-infiltrating lymphocytes and widespread expression of several immune checkpoint ligands like PD-L1, LAG-3, IDO, and CTLA4 [52, 53]. The higher mutational rate of MSI-H tumours compared with MSS group may explain these findings [21–23]. Tumours with high mutational burden have the potential to encode non-self immunogenic neoepitopes, which, in turn, activate recruitment of lymphocytes within the tumour, thus inducing an intense immune response [23, 52]. At the same time, the active immune microenvironment is counterbalanced by the expression of immune inhibitory signals that contrast tumour elimination, as illustrated in Fig. 1 [21, 22, 52, 54]. The hyper-activation of cytotoxic lymphocytes within the tumour may lead to increased apoptosis of neoplastic cells, explaining the better outcomes of MSI-H GC patients compared to the MSS subgroup [20]. The Medical Research Council Adjuvant Gastric Infusional Chemotherapy (MAGIC) trial is the first prospective study reporting an association among MSI-H, MMRD, clinical features, and survival in patients with non-metastatic GC [20, 56]. This phase III study compared the effect of perioperative chemotherapy with epirubicin, cisplatin, and 5-fluorouracil plus surgery with surgery alone, in patients with resectable gastroesophageal cancer. Considering the clinico-pathological features of patients included in the trial, female sex, older median age, Lauren intestinal subtype, low rate of metastatic lymph nodes, and stomach location were found to be correlated to the MSI-H subgroup (8.5% of all patients), compared with MSS and MSI-L classes [20]. Although none of the reported differences between MSI-H and MSS-L cases resulted significant, data from MAGIC trial are widely overlapping and confirm the main retrospective reports concerning microsatellite unstable GC [20, 42, 45, 46].

## MSI survival and response to chemotherapy in the early and advanced stages of GC

The positive association between MSI-H phenotype, MMRD, and better prognosis has been suggested in several GC studies [41, 45, 46]. Two recent meta-analyses including 17 and 21 studies, respectively, found a consistent positive effect of microsatellite unstable status on prognosis [42, 45]. Interestingly, the four different TCGA subgroups of GC [5] have been correlated to survival outcomes in another study [57]. In particular, the EBV subtype reported the best prognosis and the genomic stable (GS) subtype the worst. Microsatellite unstable subgroup and CIN are related with poor OS compared to EBV subtype but with more favourable survival rates compared with GS patients. These results were mainly attributable to the inflammatory microenvironment and immune response observed in subtypes with a better prognosis. More precisely, the immune response resulted strongly enhanced due to viral infection in EBV subtype and as a consequence of higher mutational rate in microsatellite unstable group. These events might prevent outgrowth of cancer cells and promote their apoptosis, resulting in improved OS [57]. In agreement with these results, a positive effect of microsatellite stable *vs* unstable status on survival, but restricted only to stage II disease, has been found in a retrospective trial involving 510 operated chemo-radio naïve GC, with 16% of MSI-H patients [58]. The recent post hoc analysis of the MAGIC trial first established a correlation between microsatellite status and survival in a randomized prospective study with a control group, confirming the positive prognostic value of MSI-H in GC chemo-radio naïve population [20]. Considering the group treated with surgery, OS was significantly better for patients with MSI-H than for those with MSS or MSI-L [20]. Moreover, MSI-H counteracts the negative impact of positive resection margin (R+) after gastrectomy on prognosis, as reported in a recent large retrospective study [59]. In this study, gastric cancer patients were stratified in MSI-H (26.4% of patients) and MSS (73.6% of patients) groups. Despite the presence of R+ margin status, long term-survival outcomes were reported in the MSI-H group only, with higher 3-, 5-, and 10-year disease-specific survival rates compared to MSS patients [59]. A large amount of studies attempted to define the prognostic value of MSI-H, but far less data regarding this molecular feature and response to chemotherapy are available. Retrospective Asian studies supported the hypothesis that MSI-H stage II and III GC patients do not gain any benefit from adjuvant 5-fluorouracil-based chemotherapy, whereas patients with MSS do [18, 19, 43]. This was verified also in the MAGIC trial where MSI-H patients treated with perioperative chemotherapy reported a twofold higher risk of death compared with those with MSS [20]. Recently, a prospective genomic-profiling research confirmed the MAGIC trial results and extended the predictive meaning of MSI also to the metastatic setting [60]. Metastatic oesophageal and GC samples were evaluated and 3% of patients were scored MSI-H. MSI-H tumours showed rapid disease progression on standard cytotoxic therapy with a significantly shorter progression-free survival compared with MSS patients [60]. The fast-progressing patients received the following line of treatment with anti-PD-1 antibodies (durvalumab, pembrolizumab, and nivolumab), as a single therapy or in combination with anti-CTLA4 antibodies (ipilimumab, tremelimumab). Nearly half of the patients with durable immunotherapy responses showed a higher mutational rate and an MSI-H status [60]. The detrimental effect of chemotherapy compared with the remarkable results reported in immunotherapeutic trials may be explained on the basis of the peculiar behaviour and molecular features of MSI-H tumours reported above [5, 21–23, 52]. The MAGIC trial authors focused their attention on the unexpected different outcomes between MSI-H GC and colon cancer outcomes towards chemotherapy [20]. The choice of different platinum compound administered in the MAGIC trial—cisplatin for GC—and in colorectal cancer adjuvant studies—oxaliplatin—may have influenced the results [20, 61]. In fact, in preclinical studies, *hMLH1*-deficient colon and endometrial cell lines have been reported to be resistant to cisplatin, but not to oxaliplatin [61]. Another interesting hypothesis suggested by MAGIC trial reports is based on the immune tumour microenvironment: MSI-H tumours are strongly associated with a vigorous immune infiltrate [20–23, 52], which may suppress the residual micrometastases after surgery [20]. Chemotherapy administration may have a negative effect on the immune defences, reducing the innate positive effect of the MSI-H phenotype on prognosis, whereas immune-checkpoint inhibitors may have a synergistic activity with immune response. Despite lacking of a strong validation [17–19, 42, 45], all these data suggest the future possibility of sparing unnecessary or even worse detrimental chemotherapy to MSI-H GC patients, basing the chemotherapeutic decision-making on molecular level for each patient selection [20]. The opportunity of alternative therapeutic strategies for this GC subtype, especially focused on the immune response, might be a step towards a more personalized treatment and central issue for future studies.

## Correlation among MSI, immune response, and checkpoint inhibitors: clinical implications

Targeting immune checkpoints with monoclonal antibodies has recently reached exciting goals and it represents a promising strategy for treatment of several tumours [21, 22, 62]. Among gastrointestinal cancers, the first evidences of correlation between microsatellite unstable status and PD-L1 expression were established in colorectal tumours [33, 52, 53]. The immune microenvironment in primary colorectal cancer samples from MSI-H tumours presents a high T-helper 1/cytotoxic lymphocytes infiltrate and a widespread expression of the main immune-checkpoint molecules [52, 54]. These latter are negative regulators of T cell immune function; their inhibition results in increased activation of the immune response against tumour [52]. All compartments of MSI-H cancers, including tumour-infiltrating lymphocytes (TIL), stroma, and invasive front express many of these molecules, e.g., CTLA4, PD-1, PD-L1, LAG-3, and IDO [52, 53]. In contrast, MSS tumours and their TIL show very little amount of immune checkpoints [5, 23, 52, 54]. Based on these preliminary observations, in a phase II prospective clinical trial, a group of treatment-refractory metastatic patients with MMRD colorectal cancer, MMR-proficient colon cancer, or MMRD non-colorectal cancer received pembrolizumab, an anti-PD-1 antibody [22]. MSI was found to be a significant predictor of overall response rate (ORR)—40% of ORR in MMRD colorectal cancer, 71% in MMRD non-colorectal cancer, 0% in MMR-proficient colorectal cancer and progression-free survival rate (78%, 67%, and 11% in each subgroup, respectively) [22]. Membranous PD-L1 positivity occurred only in MMRD cancers, prominently expressed on TIL and tumour-associated macrophages located at the invasive fronts of the tumour [21, 22]. The KEYNOTE-012 trial was designed to evaluate the efficacy of pembrolizumab in PD-L1 positive advanced gastric adenocarcinomas [24]. 22% of patients with PD-L1-positive tumour showed a partial response. Genomic profiling revealed an MSI-H status in 17% of all patients; among patients with MSI-H GC, 50% reached partial response [24]. Moreover, MSI-H tumours exhibited responses to immune checkpoint inhibitors, regardless of PD-L1 expression [24, 63, 64]. Results of these studies provided justification for focusing on microsatellite instability status as additional predictive biomarker of response to immunotherapy [64–66]. Pembrolizumab has been approved in 2017 by FDA for pre-treated metastatic GC patients with PD-L1-expressing tumours [66]. The drug registration is based on the results of KEYNOTE 059 trial, testing pembrolizumab on 259 patients with gastric or gastroesophageal junction advanced adenocarcinoma [67]. Among these patients, 143 (55%) expressed PD-L1. Objective responses occurred in 13.3% of patients, with response duration ranged from 2.8 to 19.4 months [67]. Considering the MSI-H group, objective responses were even more impressive, with a reported ORR of 57% [67]. The response duration ranged from 5.3 to 14.1 months [67]. The CHECKMATE 032 study assessed the efficacy of nivolumab, another anti-PD-1 monoclonal IgG4 antibody, in a PD-L1 unselected metastatic GC population [65]. Response rate of 12% with a median duration of response of 7.1 months in responders was reported. Response rates in PD-L1 positive and negative patients resulted in 18% and 12%, respectively [65]. The combination of nivolumab with ipilimumab, an anti-CTLA4 antibody, has been tested in the same setting in separate arms of the CHECKMATE 032 study, with incremental benefits in terms of response for PD-L1 positive *vs* PD-L1 negative patients [65]. An exploratory analysis of the CHECKMATE 032 evaluated response rates and OS in nivolumab arm stratifying the patients by microsatellite status [61]. Molecular profiling revealed that 28% were MSI-H. ORR was 29% in MSI-H patients, 11% in MSI-L or MSS patients, and 9% in patients with unknown microsatellite status [64]. 71%, 28%, and 26% of disease control rate were reported, respectively. MSI-H patients reached longer median OS (14.75 months) compared with the other subgroups [64]. Correlation between response rates and MMRD/MSI-H status leads to the approval of pembrolizumab for all pre-treated metastatic MSI-H tumours, regardless of primitive tumour site [55]. Probably, due to the smaller sample sizes of the studies, efficacy results of checkpoint inhibitors in MSI-H GC are not as exciting as those reported in colon cancer trials [21, 22, 24, 63–67]. However, this therapeutic strategy is based on precise histo-pathological findings and strong molecular rationale [21–23], which support administration of checkpoint inhibitors in this GC subgroup in many settings of the disease.

## Conclusions

Gastric cancer is one of the most aggressive malignancies, with high metastatic potential. Despite the strong efforts to define better curative strategies, chemotherapy and targeted drug administration did not provide the expected results and prognosis remains poor [1]. Many GC studies are now based on the evidence that patients’ survival and treatment response do not only depend on tumour staging but also on the heterogeneous molecular features of this malignancy [5, 6, 20]. TCGA first provided a systematic classification of GC, focusing on genetic profiling, defining four different subtypes with specific molecular make-up [5]. Microsatellite unstable GC represents a group of particular interest since its peculiar immunological microenvironment and response to treatment [5, 23, 52, 53]. Large meta-analyses of retrospective studies [42, 45, 46] and the MAGIC trial [20, 56] allowed to consider MSI-H GC as a separate disease, mainly associated with older age, female sex, distal stomach location, multiple gastric cancer locations, and histologic intestinal type [20, 42, 45, 46]. These studies allowed altogether identifying a prognostic and predictive meaning to this molecular subgroup, as already assessed in colorectal cancer [12, 15]. Even though MSI-H resulted associated with a better OS in chemo-naïve GC patients compared with MSS group [20], it was related to a higher risk of death and poorer outcomes when perioperative chemotherapy was administered in more advanced disease [20]. The exhaustive molecular data of Memorial Sloan Kettering Cancer Centre, consistent with MAGIC results, extend the predictive meaning of microsatellite status to the metastatic setting and support the administration of checkpoint inhibitors to heavily pre-treated MSI-H GC patients [60]. Hence, the detrimental effects of chemotherapy compared with the promising results obtained with immune-checkpoint inhibitors have been explained on the basis of the peculiar immune microenvironment described in MSI-H tumours. In conclusion, MSI-H GC shows typical histological and molecular features, defined clinical behaviour, and peculiar responses to treatments [6, 20–22, 24, 64]. These evidences suggest that it might be useful to test GC patients for microsatellite status in all the stages of disease. Further prospective studies, especially for the early stages, with a pre-planned genetic profiling of GC patients, might validate the current evidences. Despite the encouraging results, a substantial portion of MSI-H GC patients do not gain any benefit even from immunotherapy [21, 22, 24, 64]. Hence, tailored immunotherapeutic trials might be helpful to understand the interaction between immune microenvironment and molecular tumour profile, to eventually guarantee the most suitable treatment to every GC patient.
